# Advances in the research of comorbid insomnia and depression: mechanisms, impacts, and interventions

**DOI:** 10.3389/fpsyt.2025.1468212

**Published:** 2025-03-26

**Authors:** Tao Gao, Han Xiang, Qian Nan Wu, Li Shan Zhu, Wan Juan Pei, Wei Jie Fu, Tian Shu Chou

**Affiliations:** Hunan University of Chinese Medicine, Changsha, Hunan, China

**Keywords:** insomnia, depression, syndromic mechanism, vicious circle, pathophysiology

## Abstract

Insomnia and depression, both significantly impacting public health, are common psychosomatic illnesses that frequently co-occur in the same individual. Not only do these two conditions commonly co-occur, but they also exhibit a bidirectional link, where the existence of one may heighten the risk for the other. Latest research offers compelling evidence of significant overlap in biological, psychological, and sociological aspects in the comorbidity of insomnia and depression. Building on this, we aim to examine the pathophysiology of insomnia and depression, along with their comorbid mechanisms, encompassing biological routes (like genetics, HPA axis, immune-inflammatory activation, neuroendocrine regulation, microbiome alterations, and neural circuits integrating sleep and emotion regulation), as well as psychosocial routes. Consequently, proposing a self-perpetuating and mutually reinforcing “snowball effect” model of comorbid insomnia and depression, and examining corresponding preventative intervention strategies to rectify associated imbalances. Finally, this article encapsulates the challenges in this field of study and the directions for future research. Finally, the paper points out the limitations of current research (cross-sectional data being dominant, and the mechanism of multi-omics dynamics being unknown) and the future direction (longitudinal cohort combined with computational modeling to resolve temporal interactions), which will provide a theoretical basis for precision interventions.

## Introduction

Researches on the global burden of disease showed that the number of people with depression increased from 260 million in 2017 (1) to 332 million in 2021 (2). Insomnia, the second most common mental illness (3), has a global prevalence of about 10% (4), and up to 50% for patients in primary care (5). The two illnesses are often comorbid (6, 7). About 66% of people with depression also suffer from insomnia (8), and about 20% of people with insomnia show symptoms of depression (9). Epidemiological studies have unveiled a non-random association between insomnia and depression, suggesting a distinct causal relationship and shared etiological factors between the two disorders (9). Consequently, the coexistence of insomnia and depressive disorders has long been recognized, a phenomenon referred to as comorbid insomnia and depression. Recent reviews have provided an in-depth exploration of the interrelation between sleep and depression (10), the influence of sleep on depressive disorders (11), and the models of neuropsychobiological integration which interconnect research on insomnia with that on depression (12). These studies have highlighted the significant overlap between insomnia and psychological characteristics, particularly depressive symptoms (13), and have proposed that emotional distress increases the susceptibility to insomnia (14). It is essential to emphasize that under current diagnostic criteria (15), depression can present with a multitude of symptom combinations, reflecting the heterogeneity of the disorder; similarly, sleep disorders exhibit a comparable diversity. To better investigate the correlation between the two, current research on depression and sleep disorders places a greater focus on quantifiable features rather than the traditional disorder classification methods, which are limited in effectiveness (10). In tandem with these research advancements, the American Psychiatric Association (APA) has replaced the previous concepts of primary and secondary insomnia with the term “insomnia disorder” in its diagnostic manual, the DSM-5 (16). Although significant scientific progress has been achieved in the study of comorbid insomnia and depression, there is still a lack of effective measures in actual clinical applications. Therefore, this study retrospectively summarizes the current research progress in this field by searching PubMed and Web of Science, combined with manual selection of references, and it would provide a new direction to improve the research strategy and clinical paradigm of comorbid insomnia and depression, which is expected to advance the clinical practice of comorbid insomnia and depression (10).

## Incidence of insomnia and depression

Insomnia and depression have become common illnesses in daily life. Recent studies indicate that over one-third of adults are confronted with the issue of poor sleep quality (17). The prevalence of insomnia in the general population is about 10%-15% (18). Among adults, the prevalence of insomnia ranges between 10% and 20%, with 35% to 50% of individuals exhibiting symptoms of insomnia (19). According to data from the World Health Organization (WHO), approximately 3.8% of the global population is affected by depression, which accounts for about 5% of adults (4% for males and 6% for females), and 5.7% of those over the age of 60. Globally, there are about 280 million people affected by depression (20).

Insomnia is frequently comorbid with the onset of depression. Epidemiological data suggest that compared to individuals without insomnia, those with insomnia are five times more likely to develop anxiety or depression (19). People who experience persistent insomnia are at a twofold increased risk of developing depression in the coming years compared to those whose insomnia has remitted or who have undergone cognitive-behavioral therapy or pharmacological treatment (21). Individuals with chronic insomnia are 40 times more likely to develop severe depressive disorder than those without insomnia (22). Compared to the general population without sleep issues, individuals with insomnia, even without a current diagnosis of depression, have twice the likelihood of developing the condition (23). Insomnia often occurs concurrently with depression or anxiety, with a comorbidity rate of approximately 50% (21). Over 90% of patients with depression report a decline in sleep quality (22).

## Complicated relations between insomnia and depression

Existing research indicates that there is a complex interplay between insomnia and depression, where one disorder can influence the progression of the other (10). Insomnia is often regarded as a triggering factor in the development of depressive disorders, and conversely, depression can alter sleep patterns in various ways (24). Neurobiological and behavioral evidence has shown that insomnia is associated with emotional dysregulation, negative affect, and a distinct daily mood state (25). Overall, there is substantial symptom overlap between insomnia and major depressive disorder (MDD) (26). From a therapeutic perspective, studies have pointed out that treating comorbid insomnia not only improves depressive symptoms but also reduces the risk of relapse (27, 28), while antidepressant medications can also enhance the sleep quality of patients (29). Therefore, the treatment of patients with depression should not only focus on the alleviation of psychological symptoms but also address the treatment of their sleep disorders (11).

The causal relationship between insomnia and depression is complex, with some studies indicating that insomnia is a major risk factor for depression (5, 19, 30–32), and vice versa (33–35), with a positive correlation existing between the two (36). For instance, among mental illnesses such as bipolar disorder, major depressive disorder, and schizophrenia, only insomnia has a causal relationship with major depressive disorder, where the severity of insomnia is directly proportional to the risk of depression (37). Modern research considers insomnia symptoms to be a common feature of various psychiatric disorders, closely linked to depression (12). Recent meta-analyses have further confirmed previous observations that the correlation between insomnia and depression is more significant. This further emphasizes that insomnia may be a transdiagnostic phenomenon in the field of psychopathology (38).

Insomnia is a significant and independent predictor or harbinger of depressive episodes, sharing several underlying genetic, personality, and neurobiological changes with depression. If left untreated, insomnia may lead to or exacerbate depressive disorders, increase the risk of mortality, reduce the quality of life, and intensify acute symptoms (12, 29, 39, 40). Mendelian randomization analysis has reinforced the evidence for a causal relationship between insomnia and depression (13). One study (41) has hypothesized that there may be a mutually predictive relationship between insomnia and depression. Subsequent research has further indicated that insomnia and depression can predict each other (42–45). However, the improvement of insomnia symptoms may be independent of the remission of depressive disorders (46), suggesting that there may also be an uncertain causal interplay between these two disorders.

In summary, the relationship between insomnia and depression is multidimensional, involving various aspects such as biology, psychology, and therapeutics. When treating depression, it is imperative to take into account the patient’s sleep status, as improving sleep quality can not only alleviate depressive symptoms but also potentially reduce the risk of disorder relapse.

## Social impact of insomnia and depression

The impact of insomnia and depression comorbidity is extremely significant. A study in Mexico showed that the average cost of treatment related to depressive-insomnia comorbidity in the first year was $3,537.57 per patient, and the annual financial burden for patients treated in the country’s private healthcare system amounted to $293 million (47). In addition, Wickwire EM et al. found that adult insomnia patients treated for depression had a 2.2-fold increase in healthcare costs (48). In fact, a study by Liu M et al. found that depression-insomnia comorbidity significantly increased the demand for all types of healthcare resources (49). About a quarter of the global population is affected by mental dysfunction (11). As for the mood disorders, they can cause huge social and economic costs (50). In the United States, the number of people who report suffering from insomnia is estimated at 32 million, with an average annual medical cost of approximately $5,000 per patient, resulting in an estimated total social cost of insomnia of $160 billion per year (16). Insomnia increases the risk of mental disorders, medical problems, and daily life-related dysfunction in adolescents (51). There is a high prevalence of insomnia and its wide-ranging impact on quality of life, occupational functioning, and physical and mental health, all of which suggests that insomnia imposes a significant burden on the individual as well as on society (6). According to the World Health Organization, depression is predicted to be a major contributor to disability around the world by 2030 (52).

## Correlations between insomnia-depression comorbidity and other disorders

Insomnia-depression comorbidity is not only a great socio-economic burden, but may also increase the risk of other disorders. Nearly 20% of Hungarian high school students suffer from Internet use problems, which are closely related to Insomnia-depression comorbidity (53). People suffering from Insomnia-depression comorbidity are exposed to a higher risk of Alzheimer’s disorder and related dementia, as well as a higher mortality rate (54). Additionally, insomnia-depression comorbidity has been identified as a risk factor of off-hospital cardiac arrest in young adults (55). In fact, individuals with other underlying disorders are more likely to experience symptoms of insomnia- depression comorbidity. In Jordan, the prevalence of insomnia-depression comorbidity was significantly increased in female patients with multiple sclerosis stress (56). Compared to individuals without symptoms of leg motor restlessness (LMR), individuals with LMR demonstrate a higher incidence of insomnia-depression comorbidity (57). After being diagnosed with hepatocellular carcinoma, patients face a higher risk of insomnia-depression comorbidity (58). Numerous studies have suggested that insomnia may play a mediating role between depression and other disorders (59–64). It should be especially noted that suicide has become one of major causes of death globally, with approximately 1 million deaths per year worldwide (11). Insomnia-depression comorbidity is a major risk factor for suicide-related ideation and behavior (16).

## Complex regulatory mechanisms of insomnia-depression comorbidity

### Biological pathways

#### Brain

According to neuroimaging studies, patients with major depressive disorder (MDD) show significant structural and functional changes in regions of the pre-frontal cortex (PFC), anterior cingulate cortex (ACC), insulae, and limbic system (including the amygdala). These changes may be related to abnormal functional connectivity between key nodes of the salience network and the default mode network (65). In particular, insomnia patients suffering from depression had reduced volume in the orbital frontal cortex (OFC) (66). Altered functional connectivity in the striatum may increase the risk of depression and anxiety in patients with insomnia (67). However, most neuroimaging studies only assess the brain during wakefulness, and quantitative measures such as electroencephalography during sleep may provide a deeper understanding of insomnia-depression comorbidity (10). For example, measurements of sleep maps in depressed patients have revealed structural changes in sleep that typically include impairments in sleep efficiency and continuity, reductions in slow-wave sleep (SWS), and loss of REM sleep suppression (11). Overall, insomnia and depression may be distinct aspects of a single dynamic neurobiological syndrome, and common dysregulation of neural areas of the brain may contribute to insomnia-depression comorbidity (68).

#### Reward network system

When an individual’s needs and desires are satisfied, positive emotions and feelings usually arise; conversely, negative emotions and feelings may be triggered (69). From a neurobiological perspective, lack of pleasure is usually associated with dysfunction of the reward network system (70). The reward network system is mainly regulated through the VTA-NAc-mPFC circuit and multiple brain regions including OFC, HT, LHb, DS, etc., and involves a variety of neurotransmission modalities such as glutamatergic, dopaminergic, and γ-aminobutyric acidergic (71–73). Insomnia may trigger emotional dysfunction, and its mechanism may be related to the reduced ability of regions of the brain that control emotions and rewards to respond to positive emotional stimuli (74). Chronic insomnia disorder (CID) is often accompanied by depression, and both involve disruption of the reward network system. Studies have shown that patients with chronic insomnia disorder and hyperdepression (CID-HD) have reduced functional connectivity in the nucleus ambiguous in systems of the reward network, the default mode network, the salience network, and the sensorimotor network (75). Non-restorative sleep, as a manifestation of insomnia, may affect reward processing and contribute to depression by altering the activity of regions responsible for emotional control in the prefrontal cortex (76).

#### Circadian rhythm system

Circadian rhythms and sleep function are important for many disorders associated with the reward system, including depression, and a large number of animal studies have demonstrated the role of circadian rhythms and sleep function in the regulation of neural reward network system (77). Currently, a large number of data support the idea that there is an overlap between impaired regulatory mechanisms of the sleep-wake cycle and the mechanisms of depression (11). Massive longitudinal studies have shown that insomnia and late night circadian rhythm preference are significantly associated with the development of depression as its unique risk factors (78). Insomnia and depression may result in comorbidity because a joint overactive arousal system (such as overactivation of orexigenic neurons) can lead to physiological hyperarousal and emotional hyperreactivity, which disrupts the sleep-wake balance and exacerbates emotional dysregulation (12). Biological clocks monitor the passage of time using changes in the level of protein oscillations in negative feedback loops to achieve physiological and behavioral adaptation of organisms in synchrony with the Earth’s circadian rhythms (79, 80). Endogenous circadian pacemakers, as crucial determinants of wakefulness and sleep propensity over a 24-hour cycle, whose timing errors may contribute to the development of insomnia symptoms (e.g., difficulty falling asleep and problems with sleep maintenance) and insomnia (81), play an important role in circadian rhythm disruption leading to depression (24, 79). Instability of rapid eye movement (REM) sleep, defined as frequent awakenings during sleep, has been shown to be a hallmark of insomnia, depression, and anxiety and may be a pathway leading to these symptoms. In patients with depression, hyperactivity of the cholinergic system or weakening of the aminergic system may lead to an advance in REM sleep, and REM sleep instability may impede the proper functioning of emotional processing and emotional neural networks throughout the night, thereby increasing the risk of internalizing disorders (12, 68).

#### Brain-derived neurotrophic factor

Circadian rhythm changes are also significantly associated with downregulation and disturbed expression of the neurotrophic factor BDNF (29). During mild or transient acute sleep deprivation, cortical BDNF levels show upregulation, but this change gradually diminishes with prolonged sleep deprivation, manifesting as a sign of fatigue in a homeostatic phenomenon. The failure of cortical BDNF upregulation could explain the lack of NREM sleep rebound in patients with chronic sleep deprivation, a vicious cycle that may lead to nonrestorative sleep and persistent insomnia (29). Rahmani M’s study states that chronic sleep deprivation and insomnia are recognized as external stressors that can lead to depression, with biomarkers of reduced hippocampal brain-derived neurotrophic factor (BDNF) levels and disruption of BDNF expression in the frontal cortex, as well as reduced serum BDNF expression levels and impaired circadian alterations (29). Serum BDNF levels are commonly reduced in patients with major depressive disorder (MDD), and this trend toward reduction is more pronounced in individuals who also suffer from insomnia (15). Both theoretical and empirical studies suggest that BDNF may be a key mediator linking insomnia and depression (74). Research by Ballesio A and colleagues reveals that the BDNF signaling pathway may be involved in the pathology of depression and insomnia in patients with obstructive sleep apnea (75).

#### Inflammatory response

Inflammation plays an important role in adult psychiatric disorders (82). It has been noted that there are direct interactions of the immune system with the HPA axis, the afferent nervous system, and the neuroendocrine system (83). The complex interplay of cell-mediated immune activation with inflammation and its associated consequences (involving the HPA axis, vagus nerve, circadian genes, gut microbes, and BDNF, among others) may collectively contribute to neurological progression affecting specific areas of the brain, thus contributing to the concomitant occurrence of insomnia and depression. In fact, what usually occurs first in this process is the triggering of insomnia or depression, subsequently followed by the formation of a vicious cycle due to successive negative effects, which continues to be amplified through a snowball effect, ultimately leading to the phenomenon of insomnia-depression comorbidity. Considering the insomnia perspective, sleep disorders may cause activation of the sympathetic nervous system and β-adrenergic signaling, which leads to the release of neuromediators and NF-κB-mediated inflammatory responses. Meanwhile, chronic inflammation may be induced through microglia and astrocytes, which may lead to overactivation of the stress system, and the changes in the activity of the inflammatory factors may cause the accumulation of neurotoxic proteins and oxidative stress, which results in impairment of the central nervous system, potentially exacerbating neurodegeneration and progression in mood disorders and psychiatric disorders (68, 84, 85). Considering the depression perspective, several meta-analyses have confirmed that proinflammatory cytokines and acute phase proteins are increased in patients with major depressive disorder (MDD) (86). Patients with depression manifest increased brain inflammatory activity, elevated levels of peripheral pro-inflammatory cytokines, and perturbed gene expression of associated inflammatory regulatory networks (71). Besides, peripheral inflammatory molecules can cross the blood-brain barrier through the transport system or enter the nervous system through leaks in periventricular structures (e.g., subforaminal organs, posterior regions, endplate vascular organs, median eminence, pituitary gland, and pineal gland), and can affect BDNF signaling, nuclear factor-κB signaling pathway, and NMDA receptors, which may lead to decreased neurogenesis and cell proliferation and increased excitotoxicity. They can also have an effect on inflammatory molecules in the brain through stimulation of the vagus nerve, which can directly or indirectly affect sleep regions of the brain (75, 87, 88).

#### Microbiome-gut-brain axis

The adult microbiome consists of more than 1,000 species and 7,000 strains (89). Symbiotic microbial stimulation can promote maturation of the immune system, while microecological dysregulation due to gut barrier damage can facilitate microbial interactions with host immune cells (90). The gastrointestinal tract serves as a key endocrine organ, and gut bacteria can interact with the enteric nervous system and the central nervous system (91, 92). Recent studies have pointed out that the gut flora plays an important role in the regulation of circadian rhythms and that changes in circadian rhythms can influence the structure of microbial communities and metabolic processes (93). Interestingly, the gut microbiome also appears to play a role in the production of brain-derived neurotrophic factor (BDNF) (83).

Changes in gut microbiota have been reported in patients suffering from sleep disorders and neuropsychiatric disorders (94). Disruption of the gastrointestinal microbiome may play a key role in the development of psychiatric disorders such as insomnia and depression. Clinical studies have shown that the gut microbiome is able to modulate sleep and psychoemotional states of the host through the microbe-gut-brain (MGB) axis (95, 96). For example, Dorea bacterium under the thick-walled phylum Trichosporonaceae has a correlation with depression and sleep quality (97, 98). A possible mechanism for this process is that dysregulation of the gut microbiome (e.g., changes in microbial composition and metabolism, damage to the intestinal barrier) influenced by internal and external factors triggers an inflammatory response, which may lead to aberrant immune responses and metabolic disorders, thereby affecting neurological function (including changes in the metabolism of neurotransmitters), which triggers or exacerbates insomnia and depression, and ultimately creates a vicious cycle.

#### Hypothalamic–pituitary–adrenal axis

Under the influence of a variety of factors, the HPA axis may become overactive, leading to elevated levels of CRH and CORT, which in turn triggers organismal responses such as immune-inflammatory responses and gut microbiome imbalances, as well as changes in specific areas of the brain. In the case of glucocorticoid resistance, the immune-inflammatory response can be further augmented and exacerbate neuroinflammation, and these changes in turn exacerbate the HPA axis load, potentially leading to or exacerbating insomnia and depression in a vicious cycle. This view is further supported by relevant evidence, such as the finding of elevated levels of cardiac rheumatoid hormone (CRH), adrenocorticotropic hormone (ACTH), and corticosterone (CORT) in the morning serum of patients with depression-comorbid insomnia (DCI) (99). In addition, the main role of glucocorticoids is to redistribute energy resources and to promote the restoration of homeostasis and defense mechanisms in the body after intense activity (24). Inadequate or over-expenditure of energy supply may lead to debilitating and degenerative neurological functions, which in turn may affect insomnia or lead to depression (52).

### Psycho-sociological pathways

The scientific point of view regards the brain as an organ that regulates mental activity and emphasizes that the mind is a manifestation of brain function, i.e., a subjective and dynamic reflection of objective reality by the brain. In fact, the biological basis of mental activity covers the nervous system, the endocrine system, and genetic factors. The cerebral cortex, as a part of the nervous system, is the core material basis for the generation of psychological activities, and the organism relies on the nervous system to accomplish muscle movement, sensation, autonomous activities and hormone secretion. Psychological factors influence physiological and pathological processes in the body through the hypothalamus-pituitary-hormone (HPH) system. Broadly speaking, when psycho-behavioral and/or socio-environmental factors act, different areas of the brain become active accordingly. On the one hand, these activities stimulate the HPH system to produce hormones that act on the internal organs and the autonomic nervous system; on the other hand, the hormones have an excitatory or inhibitory effect on nerve cells through the blood circulation. Research has shown that psychological traits such as personality, temperament and abilities, as well as patterns of human behavior, are associated with genetic factors. Psychological activity has not only a biological basis, but also a social basis; that is, the psyche is a product of socialization formed under the influence of material and cultural conditions (69).

Individuals with low self-awareness (e.g., deficits in responsibility, motivation, and self-control) and emotional instability (e.g., worrying, repetitive thinking, poor coping, and diminished emotional regulation) are prone to cognitive, emotional, and behavioral deficits. These deficits contribute to negative cognitive activities, such as excessive worrying and repetitive thinking related to sleep, which in turn lead to insomnia. Patients may exhibit depression symptoms when they become aware of the day-to-day consequences of insomnia, such as difficulties in coping socially and in daily life, as well as a decreased sense of self-awareness and emotional stability in dealing with these challenges (100). Recent studies have shown a positive correlation of conspiracy theory mindset with insomnia and psychological distress (e.g., anxiety and depression) occurring one month later (101). Dramatic social upheaval is an important causative factor, especially during COVID-19, when symptoms of insomnia and depression were prevalent among people from different occupations around the world (102–108). Generally, insomnia may reduce an individual’s participation in positive social environments. For example, intensifying feelings of failure and worry about the future, lead to an individual feeling overburdened on a physical and cognitive level when coping with challenges in social and family environments, which in turn may impair interpersonal relationships and ability to cope with stress, increase the likelihood of stressful life events and adverse reactions to them, and thus increase the risk of depression (9, 68).

Depression may lead to a decrease in daytime social activities and reduce daylight exposure, as well as concentration of thoughts at night leading to difficulties in relaxation and increasing nocturnal activity, which disrupts circadian rhythms and sleep-wake patterns, further exacerbating insomnia symptoms. Stressful life events may promote the development of depression in individuals (109). To some extent, depression can be seen as an adaptive strategy that helps individuals cope with the challenges of uncertainty and unfavorable relationships in the social world (110). Not all individuals who experience stress in early life develop the disorder in subsequent trauma or stress, and similarly, not all adults with depression have experienced early life stress (24).

**Figure 1:**
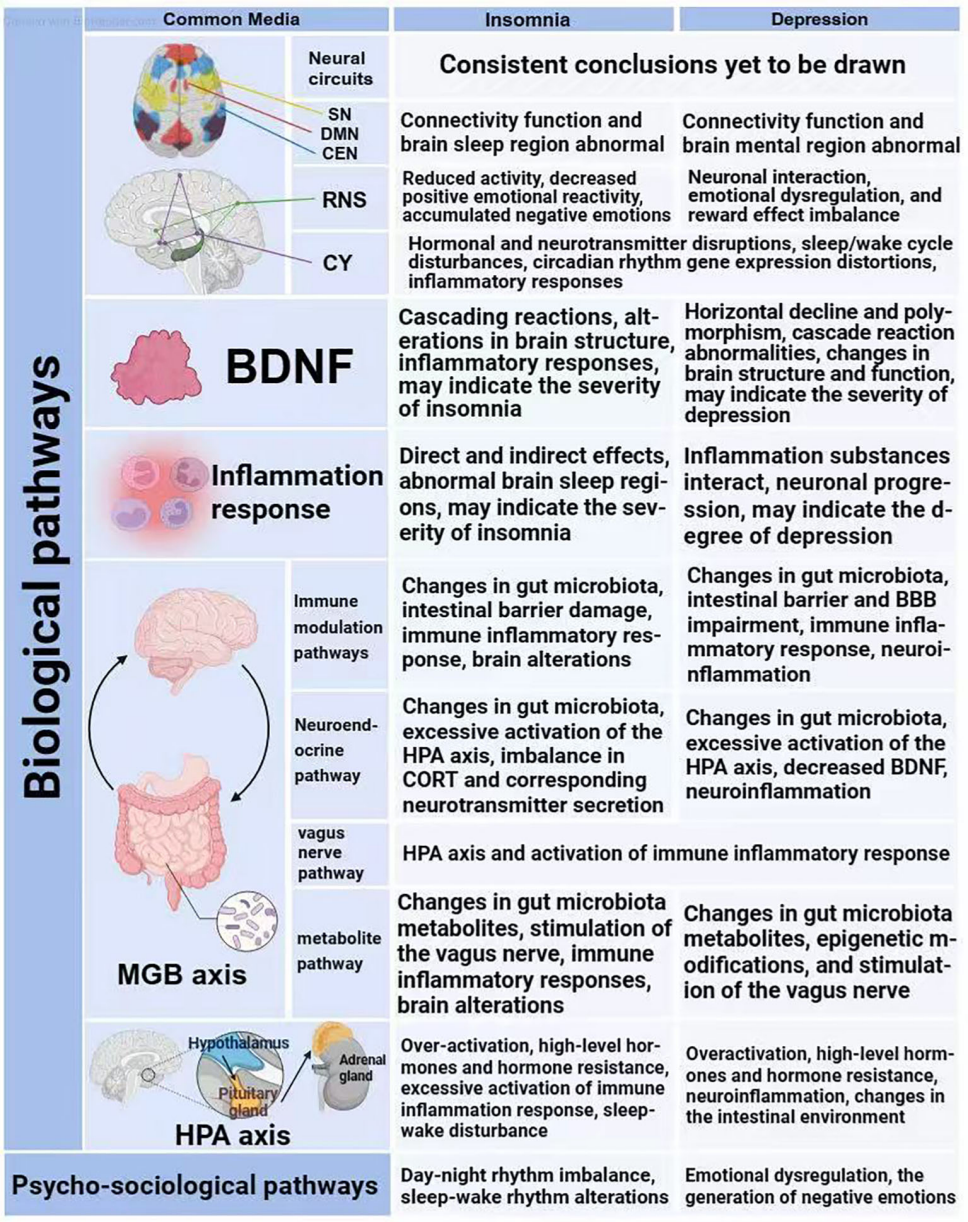
The bio-psycho-social basis of the snowball effect model.

**Figure 2:**
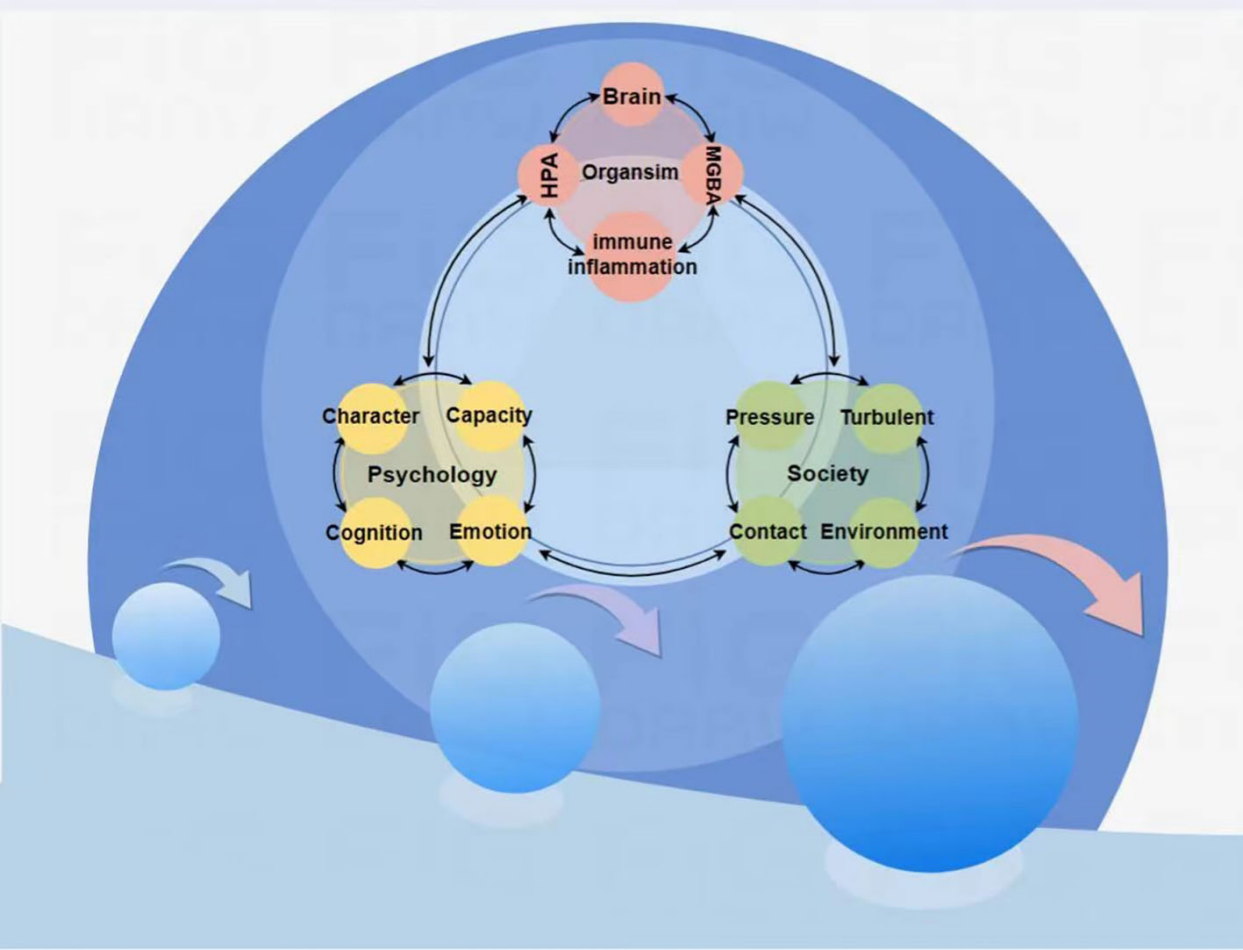
Pathophysiologic assumption of insomnia-depression comorbidity pathway.

**Figure 3:**
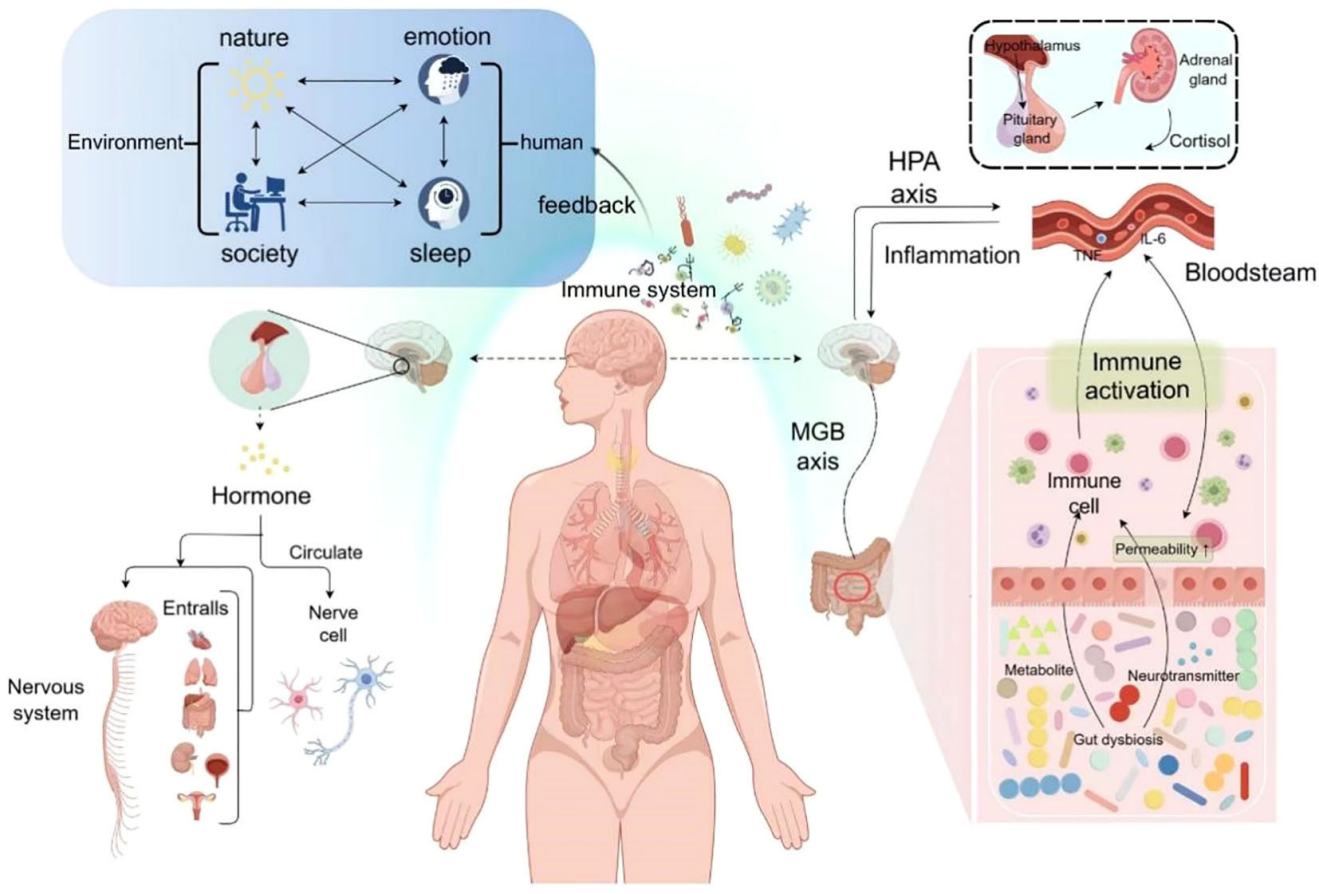
Insomnia-depression comorbidity mechanism.

In summary, the pathophysiological mechanisms of insomnia and depression are highly overlapping (See **Figure 1**), based on similar mechanisms occurring in the same organs and tissues. Regardless of the specific pathogenesis of the comorbid disorders, once the two disorders coexist, they may maintain or even exacerbate each other, creating a vicious cycle to the extent that the presence of one disorder may hinder the recovery of the other. Therefore, this review proposes a snowball model of the interaction between insomnia and depression (See **Figure 2**), and this model conceptualizes the process of interaction between the comorbid disorders of insomnia and depression. The first feature of this model is that the various nodes and connections involved in the model, despite deviations, ultimately converge on a common pathway for insomnia and depression, which is centered in the brain; the second feature of this model is that the two disorders interact with each other and can reinforce each other; the third feature of this model emphasizes that the mechanisms behind insomnia and depression are more similar than different; and the fourth feature of this model involves the imbalances in the trinity (biology, psychology, and society) (14). This common mechanism can be summarized as a combination of self-maintenance and mutual reinforcement, leading to biological, psychological, and social imbalances that ultimately trigger brain dysfunction. (See **Figure 3**).

For the snowball model of the pathogenesis of insomnia-depression comorbidity, we have divided it into three stages. Small snowball stage: mild internal or external stimuli can lead to dysfunction of the more fragile and susceptible systems in the body, further affecting other systems, and these dysfunctions ultimately affect the pathways in the brain that regulate insomnia and depression, leading to abnormalities in their function and/or structure, thus triggering mild insomnia-depression comorbidity. This phenomenon may reflect a protective response of the organism that is closely related to the individual’s ability to self-regulate. For example, the initial response of the immune system to minor internal or external stimuli activates a mild inflammatory response, causing mild dysfunction of the MGB and HPA axes, as well as minor fluctuations in neurotransmitters, hormones, and other substances, which ultimately leads to malfunctioning of the brain in the regulation of sleep and psychosomatic functioning, which leads to mild insomnia-depression comorbidity. This is not only a unidirectional process of cascading amplification, but each link contains feedback mechanisms that lead to the cumulative character of multiple vicious circles - a combination of self-sustaining and mutual reinforcement, which is not evident in the initial stages. Medium snowball stage: more intense internal or external stimuli lead to dysfunction in highly susceptible and vulnerable systems in the body, which further spreads to other systems, and these dysfunctions affect pathways in the brain that regulate insomnia and depression, triggering abnormalities in their functioning and/or structure, which leads to the onset of a moderate insomnia-depression comorbidity. This condition may be based on a small snowball effect, where one of the links interacts and accumulates with the other cycles, leading to an overall exacerbation of the problem. As an example, the initial response of the immune system is activated by a stronger stimulus, triggering a heavier inflammatory response, which leads to a moderate dysregulation of the MGB and HPA axes, as well as significant changes in the production of neurotransmitters, hormones, and other substances, which ultimately leads to malfunctioning of the brain in the regulation of sleep and psycho-social functioning, and triggers the onset of the moderate insomnia depression comorbidity. Self-sustaining and mutually reinforcing features are evident in this stage. Snowball stage: Extreme internal or external stimuli lead to dysfunction in highly susceptible and vulnerable systems of the body, and these dysfunctions further spread to other systems, resulting in functional and/or structural abnormalities in the brain’s pathways that regulate insomnia and depression, leading to the onset of severe insomnia-depression comorbidity. Similarly, this may be based on a meso-snowball effect, resulting from the interaction and accumulation of various components. Taking the initial response of the immune system as an example, extreme stimuli activate the immune system, triggering a severe inflammatory response, which leads to heavy dysregulation of the MGB and HPA axes, as well as significant changes in the production of neurotransmitters, hormones, and other substances ultimately dysfunctionalizing the brain in the regulation of sleep and psychosomatic functioning, leading to the onset of the major insomnia depression comorbidity. Self-maintenance and mutual reinforcement characterize this stage significantly.

### Theoretical innovations: comparison with classical models

Based on classical theory, the snowball model of this study achieves mechanism integration and dynamicity expansion. The comprehensive insomnia-depression bi-directional model, proposed by Riemann (12), systematically integrates Predisposing, Precipitating, and Perpetuating factors, but it is a linear phased framework that focuses on the segmentation of mechanisms, while this study constructs a non-linear dynamic system model, enters feedback loops among multiple maintenance factors (e.g., microbiome-gut-brain axis and inflammatory response), and reveals the combined self-sustaining and mutually reinforcing mechanisms of the Trinity imbalance. In addition, the model further reveals biological mediators compared to Fernandez-Mendoza’s Longitudinal Study of Insomnia-Depression (111), which focuses on behavioral and psychological mediators (e.g., coping ability). Notably, the snowballing model in this study is consistent with the RDoC concept (112), emphasizing the integration of multilevel data to identify tipping point effects. In the future, RDoC matrix design can be used to analyze the dynamic mechanism of comorbid insomnia and depression from the gene-circuit-behavior linkage perspective, and to develop multi-dimensional and multi-target preventive intervention strategies.

### Prevention and intervention for insomnia-depression comorbidity—a personalized prevention intervention model

The link between insomnia and depression emphasizes the importance of preventing both disorders and the need for early intervention, which should be a focus of public health and clinical care. The paper model the complex regulatory mechanisms of insomnia-depression comorbidity by summarizing the results of existing studies, which could be valuable for the prevention and treatment of this comorbidity.

**Figure 4:**
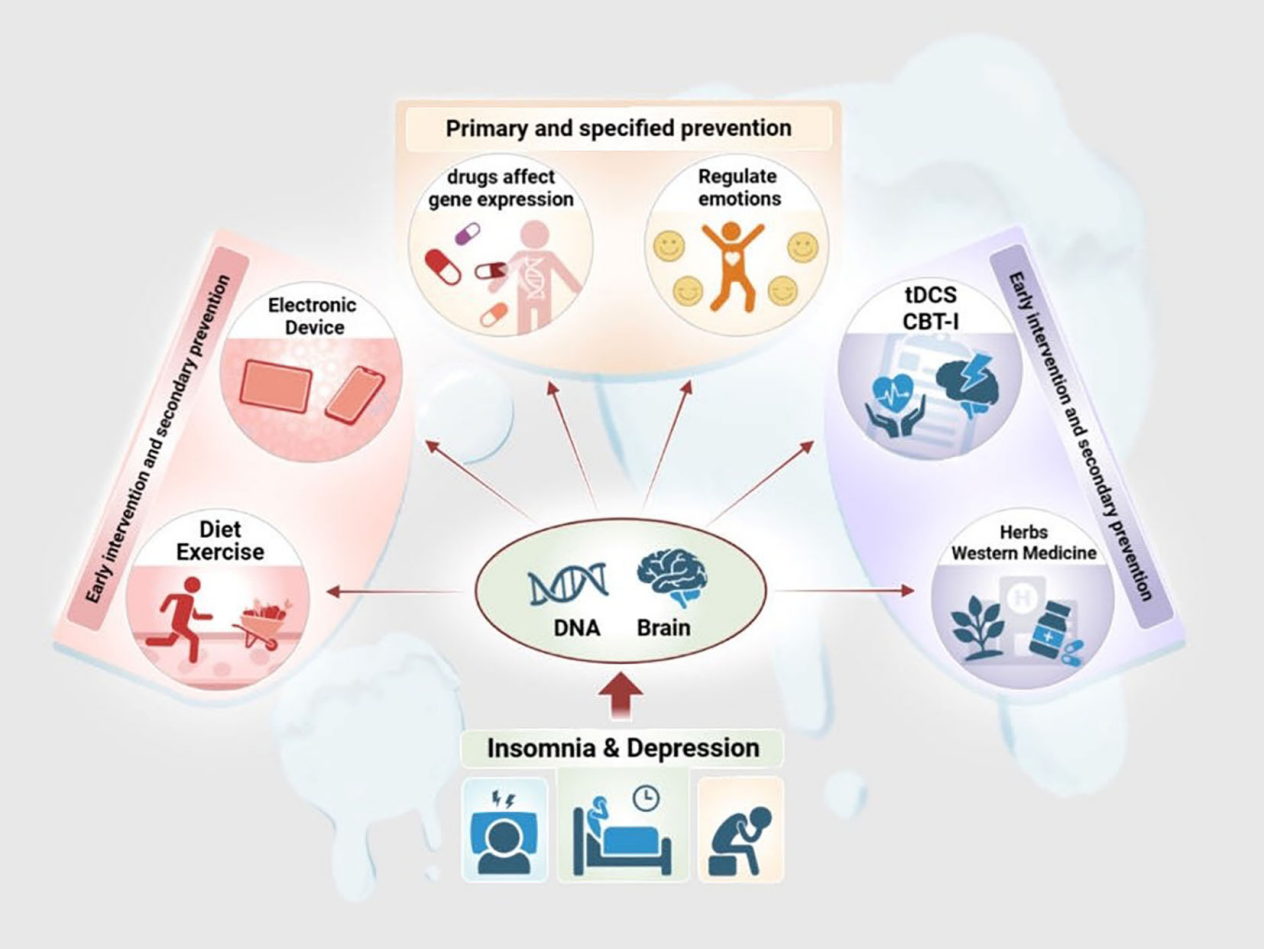
Insomnia-depression comorbidity treatment mechanisms.

This review describes the causative mechanism of insomnia-depression comorbidity as a multifunctional psychosomatic disorder centered on the brain and characterized by a combination of self-sustaining and mutual reinforcement. Importantly, insomnia and depression may occur concurrently or sequentially and may differ in degree, that is, insomnia may also be only a consequence of depression and vice versa. However, dual-targeted interventions for insomnia and depression are likely to be effective in both primary and secondary cases, and the review therefore proposes a personalized preventive intervention model for insomnia-depression comorbidity (See **Figure 4**).

Preventing the snowball from forming – focusing on prevention (primary prevention and targeted prevention); Alleviating the progression and facilitating the dissipation of the snowball – mainly via intervention (small snowball: early intervention and secondary prevention; medium and large snowballs: mid and late stage of prevention and tertiary prevention); Additionally, some early intervention and secondary prevention measures are also applicable to prevent the snowball from forming. In general, the optimal treatment method might involve restoring balance to the biological, psychological and social imbalances.

**Figure 5:**
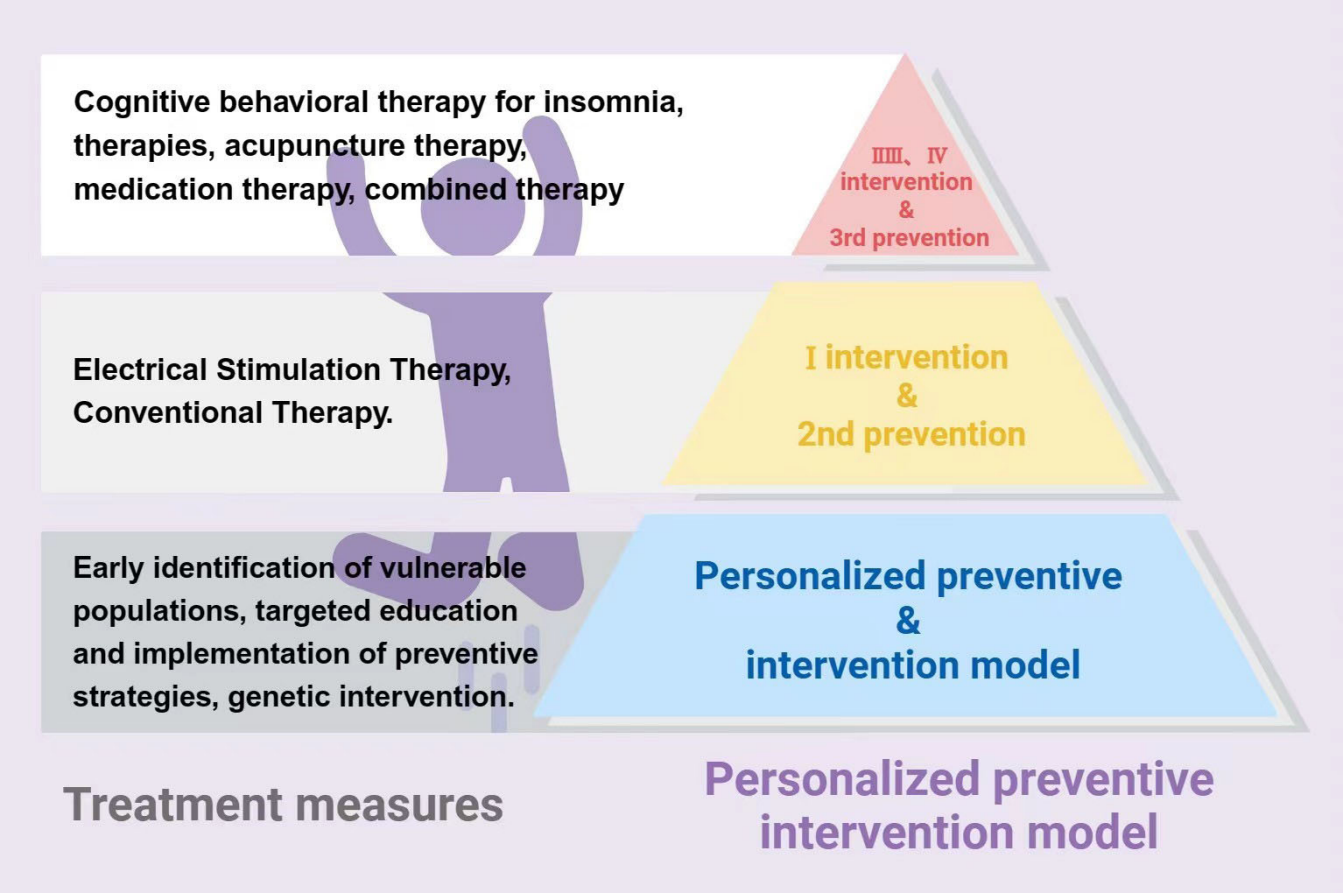
Insomnia-depression comorbidity treatment.

In the final part, the review will look at potential approaches of primary prevention of insomnia-depression comorbidity, prevention of depression in patients with insomnia, as well as potential approaches to early intervention, secondary prevention, mid-late intervention, and tertiary prevention in patients with depression and insomnia. The review suggests that key factors such as active late nights and unhappy events should be targeted for primary prevention of insomnia-depression comorbidity; that factors such as depressed mood, loss of interest, and nonrestorative sleep should be the focus of early intervention, and changes like ones in appetite and sleep can be considered as indicators of secondary prevention; that typical symptoms of insomnia and/or depression (e.g., difficulty in falling asleep, decreased quality of sleep, decreased sleep duration, long periods of low mood or loss of pleasure, daily fatigue, feelings of guilt or worthlessness) indicate the need for interventions in the middle to late stages; symptoms such as panic palpitations, disorganized thinking, and even suicidal thoughts may point to the need for tertiary prevention (79). (See **Figure 5**) In this process, the review emphasizes comprehensive, multidimensional and integrated consideration of the patient (including but not limited to the patient’s condition, willingness to be treated, financial ability and the safety, efficacy, risk and other possible subjective and objective factors of the treatment plan) to select the best plan suitable for different patients and to achieve shared decision-making between doctors and patients (see **Figure 5** for details) While the review advocates comprehensive consideration of the patient’s situation, it also emphasizes identifying and targeting measures to the core of the disorder, as well as taking into account other relevant underlying disorders, in order to maximize the benefits and minimize the risks. Notably, clinicians should understand the important differences between current practice and evidence-based guidelines (21). In addition, the review lists challenges and future research questions in the field.

## Key challenges and future perspectives

Though the present model has advantages in mechanism integration, its validation needs to address core issues such as differences in temporal resolution of cross-scale data, parameter estimation of nonlinear interactions, and statistical identification of bidirectional causation. For example, the temporal association between microbiome and neuroimaging may be confounded by confounding factors such as diet and antibiotic use. The biopsychosocial interactions may be difficult to capture in a single study design. Similar to the Network Neuroscience approach (113), future researches need to combine rigorously controlled longitudinal designs with causal inference statistical methods, by using multi-layer network models (e.g. defining the diversity of the flora as the biological layer, the functional connectivity of the resting state as the neural layer, the social support scores as the behavioral layer), computational modeling (e.g. System Dynamics Models and Bayesian Networks), and multimodal data integration (e.g. combining functional magnetic resonance imaging, polysomnography, blood biomarkers, microbiome analysis, social network analysis) to quantify the strength of cross-level interactions, and combined with cross-lagged designs, intervention trials (e.g. probiotics and social skills training) to validate the dynamic plasticity of the model. Notably, Network Theory of Mental Disorders (114–116) proposed that dynamic interactions between symptoms may be a key pathway for the expression of multisystemic imbalances at the behavioral level.

Previous research has made significant progress in understanding and treating insomnia and depression. Nonetheless, our understanding of the etiology, pathogenesis, pathophysiology, biomarkers, and optimal treatments for insomnia and depression and their comorbidities remains limited. Therefore, it is necessary to advance mechanistic research through more refined study designs (e.g. multi-level data integration, causal inference methods) and to validate the value of clinical applications through multi-center large-sample studies. These future directions include:

1. Assessing the unique impact of comorbidity on quality of life and its differences from anxiety and pain.
2. Analyzing the association of insomnia and depression-related parameters with specific symptoms and disease course.
3. Uncovering the temporal dynamics and interactions of biopsychosocial mechanisms.
4. Integrating multimodal data from neuroimaging, immunoinflammatory indexes, and microbiomes, and constructing multilevel interaction models.
5. Quantifying the impact of demographic and environmental factors on the quality of life of patients.
6. Developing precise quantitative tools to assess sleep-psycho-environmental indicators, which can be combined with multilayer network models and system dynamics modeling to improve measurement accuracy.
7. Establishing a personalized intervention system based on biopsychosocial characteristics, and integrating time-varying network modeling and real-time biomonitoring techniques to analyze multi-system dysfunctions by the intrinsic mechanism of network solidification driven by dynamic interaction of symptoms can guide the precise optimization of dynamic intervention strategies.
8. Promoting the translation of evidence-based therapies into clinical practice, and enhance the standardization of diagnosis and treatment through interdisciplinary collaboration.

## Summary

This review summarizes research advances in the mechanisms, effects and interventions for insomnia, depression and their co-morbidities. Insomnia and depression may be both independent and interacting disorders, or they may simply be different symptomatic manifestations of the same underlying process. Common processes across multiple biological systems suggest that understanding these phenomena may require a systematic approach. These processes are, in part, components of adaptive systems that interact with psychological and social/environmental factors. This suggests that insomnia-depression comorbidity may arise from a continuum, parallelism, and interplay of biological, psychological, and social mechanisms, i.e., disruptions in any of the factors in the matrix may cause physiological and behavioral dysregulation of the brain, which in turn leads to insomnia and depression. Thus, the review proposes a self-sustaining and mutually reinforcing snowball-style model to explain the underlying mechanisms of insomnia-depression comorbidity. This model innovatively integrates the dynamic interaction mechanism of multiple maintenance factors and provides a theoretical framework for analyzing pathological state transitions driven by critical effects. These risk mechanisms are expected to be feasible targets for preventive interventions that can be targeted to reduce the risk of developing other comorbidities (e.g., suicide, obesity, pain, and cardiovascular disease), thereby reducing the burden on patients and society.

Although the snowball model in this study has advantages in mechanism integration, its empirical validation needs to address technical bottlenecks such as cross-scale data heterogeneity and nonlinear interaction parameter estimation. Future studies should combine multilayer network modeling with causal inference methods, as well as focusing on the individualized intervention window period. These advances will promote the transition of prevention strategies to multi-system homeostatic regulation and achieve precise interventions based on tipping point prediction. Although this review has constructed a theoretical model through systematic literature analysis, it still needs to be further validated for its scientific validity and clinical application value through rigorously designed longitudinal cohort and computational modeling studies. At the same time, it cannot be ignored that the breadth of the topic limits the review of the entire literature on the subject.
